# Self-Perceptions of Readiness to Use Electronic Health Records Among Medical Students: Survey Study

**DOI:** 10.2196/17585

**Published:** 2020-06-12

**Authors:** Lina Lander, Sally L Baxter, Gary L Cochran, Helena E Gali, Kristen Cook, Thomas Hatch, Regan Taylor, Linda Awdishu

**Affiliations:** 1 Department of Family Medicine and Public Health University of California San Diego La Jolla, CA United States; 2 Department of Biomedical Informatics University of California San Diego Health La Jolla, CA United States; 3 Shiley Eye Institute and Viterbi Family Department of Ophthalmology University of California San Diego La Jolla, CA United States; 4 College of Pharmacy University of Nebraska Medical Center Omaha, NE United States; 5 School of Medicine University of California San Diego La Jolla, CA United States; 6 Information Services Department University of California San Diego Health La Jolla, CA United States; 7 Department of Internal Medicine University of Nebraska Medical Center Omaha, NE United States; 8 Skaggs School of Pharmacy and Pharmaceutical Sciences University of California San Diego La Jolla, CA United States

**Keywords:** electronic health record, medical student, education, training, residency

## Abstract

**Background:**

Although several national organizations have declared the ability to work with electronic health records (EHRs) as a core competency of medical education, EHR education and use among medical students vary widely. Previous studies have reported EHR tasks performed by medical students, but students’ self-perceived readiness and comfort with EHRs are relatively unknown.

**Objective:**

This study aimed to better understand medical students’ self-perceived readiness to use EHRs to identify potential curricular gaps and inform future training efforts based on students’ perspectives.

**Methods:**

The authors deployed a survey investigating self-perceived comfort with EHRs at 2 institutions in the United States in May 2019. Descriptive statistics were generated regarding demographics, comfort level with various EHR-related tasks, and cross-institutional comparisons. We also assessed the impact of extracurricular EHR experience on comfort level.

**Results:**

In total, 147 medical students responded, of which 80 (54.4%) were female, with equal distribution across all 4 years of training. Overall confidence was generally higher for students with longer extracurricular EHR experience, even when adjusted for age, gender, year of training, and institution. Students were most comfortable with tasks related to looking up information in the EHR and felt less comfortable with tasks related to entering new information and managing medications. Fourth-year students at both schools reported similar levels of comfort with EHR use, despite differences in preclinical EHR training. Open-ended comments emphasized the value of experiential training over didactic formats.

**Conclusions:**

Information entry and medication management in the EHR represent areas for future curricular development. Experiential training via extracurricular activities and early clinical exposure may be high-yield approaches to help medical students achieve critical EHR competencies.

## Introduction

Electronic health records (EHRs) have become widely adopted across the United States [1-4]. EHR use has become an increasingly prominent component of physician work time and effort across multiple specialties, in some cases equaling or surpassing time spent in face-to-face interactions with patients [5-12]. This widespread integration of EHRs has generated a national discussion regarding the role of EHR training in medical school curricula. In response, several national organizations, such as the Association of American Medical College (AAMC) [13], the Alliance for Clinical Education [14], and the Liaison Committee on Medical Education [15], have issued guidance stating that the ability to work in an EHR is a core competency for medical students before beginning residency. In fact, 2 of the AAMC’s core entrustable professional activities for entering residency relate to EHR use: (1) *enter and discuss orders and prescriptions* and (2) *document a clinical encounter in the medical record* [16]. The need for medical student preparation and readiness in EHRs has also been echoed by professional medical societies, such as the American Medical Association [17], the American College of Surgeons [18], and the American Academy of Family Physicians [19].

Despite the recognized need for directed EHR training during medical school, EHR education and use among medical students vary widely among different institutions and clerkships [20-24], and a lack of focused initiatives to engage students in EHR education may leave gaps in the competencies expected of residents [25]. Previous studies have focused on the types of tasks performed within the EHR by medical students during their clinical rotations, such as accessing information, entering information, entering notes, and entering orders. However, knowledge about medical students’ self-perceptions of their comfort and readiness in using EHRs as well as how this sense of readiness may relate to varying curricular approaches is lacking.

To fill this gap, we deployed a survey to medical students at 2 different institutions to gain an understanding about self-perceptions of EHR readiness. We hypothesized that perceived readiness would be higher among students with prior extracurricular EHR experience as well as among students who participated in an integrated EHR curriculum. The purpose of this study was to better understand medical students’ perceived readiness for EHR use and identify gaps in curriculum and training that could be addressed to improve medical school curricula and address this critical competency of EHR education.

## Methods

### Study Population

Eligible participants included all medical students from the University of Nebraska Medical Center College of Medicine (UNMC) and the University of California San Diego School of Medicine (UCSD). Currently enrolled medical students at all levels of training were eligible. Both medical schools are affiliated with academic medical centers that use the same EHR vendor. The institutional review boards of both UNMC and UCSD approved this study.

### Curricula and Electronic Health Record Training

UNMC is a 4-year Doctor of Medicine degree–granting program, affiliated with Nebraska Medicine, which enrolls approximately 135 students per year. Students acquire EHR skills throughout the 4-year curriculum. Medical students begin learning to use the EHR during the first week of school, and formal training continues over the first 18 months before entering clerkships. The UNMC preclinical phase consists of 10 organ systems–based blocks, each containing an EHR exercise. These range from a scavenger hunt, in which students learn where to locate information in the health record, to specific cases designed to help them learn order entry or how to type notes in the EHR. All preclinical exercises are performed in the EHR training environment and are supervised by a faculty member. Early sessions also have information technology staff support to help troubleshoot issues with access or functionality. The initial sessions are used to learn navigation skills—finding specific patient information, laboratory and imaging results, or searching encounter notes and discharge summaries. Once basic skills are established, EHR-based cases are used to improve students’ skills in the medical record to include documenting clinical encounters; entering orders; documenting medical history, allergies, and medications; reviewing pertinent medical information from prior notes; and using alerts and reminders in the EHR to complete health care maintenance tasks. Formal preclinical EHR training was initiated in 2017. During the preclinical phase, students also have the opportunity to practice their skills at the student-run free clinic (SHARING Clinic). Approximately 50% of the preclinical students volunteer for this clinic, which uses the same EHR as the main medical campus. Immediately before clerkships, students have a session to learn shortcuts offered within the EHR—specifically, using templates for note writing. These training exercises are designed to increase competence and confidence with the use of EHR and to promote active participation in the delivery of care during clerkships.

UCSD is a 4-year Doctor of Medicine degree–granting program, affiliated with the UC San Diego Health, which enrolls approximately 134 students per year. EHR exposure is limited in the preclinical years. There is no formal EHR training in the preclinical curriculum, unless a student elects to participate in the student-run free clinic. Approximately 80% of first- and second-year students volunteer at the student-run free clinic, where students use a clinic-specific EHR from the same vendor as the academic medical center. Students who volunteer in the free clinic undergo a 2-hour orientation session on EHR functionality and clinic-specific workflows. All medical students, irrespective of free clinic participation, receive formal EHR education during a clinical transition week at the beginning of the third year. Students review several web-based modules provided by the vendor and participate in one 2-hour didactic session that covers both inpatient and ambulatory clinic tools and workflows. Examples of skills taught include finding patients on a clinic schedule and navigating summary reports, demographics, patients’ problem lists, notes, and labs.

At both institutions, third- and fourth-year medical students are actively engaged in EHR use in their clinical clerkships and elective rotations. Both institutions also require subinternships where students function as interns under the close supervision of faculty physicians and senior residents. EHR tasks include placing orders throughout the hospitalization; performing medication reconciliation; and writing admission notes, progress notes, and discharge summaries. However, the main contrast in curricula is that UCSD has little formal EHR training during the preclinical years, whereas UNMC has integrated EHR training throughout the preclinical curriculum.

### Survey

We modified EHR competency assessment tools provided by the EHR vendor for formal training sessions to develop the self-perceived readiness survey (full survey instrument available in Multimedia Appendix 1). Instead of asking students to perform specific tasks such as finding allergies, immunizations, and others, we rephrased the questions to ask about self-perceived comfort levels while performing each task. Students rated the comfort level with using various EHR components on a 5-point Likert scale, ranging from 1 (very uncomfortable) to 5 (very comfortable). We also asked students to provide their gender, age group, year of training, and content and length of preclinical extracurricular EHR experience. The survey concluded with an open-ended item asking students for general comments about their EHR training and preparedness to work in the EHR. In total, 3 School of Medicine faculty members and 2 medical students from both institutions assessed the survey for face validity, readability, and understanding.

The electronic survey was administered anonymously via email to all current medical students in May 2019 at UCSD and in July 2019 at UNMC, with 2 reminder emails at 7 and 14 days after the initial invitation. Owing to the timing of the survey administration at UNMC, preclinical students were students between years 1 and 2 of the curriculum; no incoming first-year students were surveyed, as they had not yet started the curriculum. The survey remained open for a total of 30 days. Survey completion required approximately 10 minutes and did not affect students’ grades or evaluations. Survey data were collected using Qualtrics (Qualtrics, Provo, UT, USA).

### Statistical Analyses

Statistical analysis consisted of descriptive statistics using the mean and SD or counts/frequencies where appropriate. To compare categorical data between institutions, we used the Pearson chi-square test for independence. We used the Student *t* test to compare mean Likert scores for survey items. Although Likert scale data are classically analyzed with nonparametric testing such as the Mann-Whitney-Wilcoxon test, we chose to compare mean Likert values using the Student *t* test [26] to facilitate data interpretation. For any *t* test that generated a *P* value of less than .10, we conducted the Mann-Whitney-Wilcoxon test as a sensitivity analysis. For clarity, only *P* values from *t* tests are reported. In all cases, we reached the same conclusion regarding statistical significance, regardless of whether we used a *t* test or a Mann-Whitney-Wilcoxon test. For all hypothesis tests and models, statistical significance was defined as a *P* value of less than .05. Statistical analyses were conducted using Stata version 11 (StataCorp LLC, College Station, TX, USA) and R (RStudio Inc, Boston, MA) [27].

## Results

### General Demographics

In total, 147 medical students responded to the survey on EHR readiness at the 2 institutions. Of the 506 medical students who received survey invitations at UCSD, 95 (19%) responded. The response rate at UNMC was 13.4% (52/386). About half of the respondents were female (80/147, 54.4%; Table 1). The majority (27/52, 52%) of respondents at UNMC were aged <25 years, whereas the most well-represented group among UCSD respondents were those in the 25 to 27 years age range (*P*=.02). The gender distribution of the survey respondents was generally consistent with the overall enrollment at the 2 institutions—the proportion of females in the overall UCSD student population was 52.7% (369/700), whereas at UNMC, it was 45.0% (175/389). The age distribution of the survey respondents at UCSD corresponded with that of the overall student population (188/700, 26.9% aged <25 years; 291/700, 41.6% aged 25-27 years; and 221/700, 31.6% aged ≥28 years). The survey respondents at UNMC had a greater proportion of individuals younger than 25 years (27/52, 52% of the survey respondents compared with 104/389, 26.7% in the overall student population).

**Table 1:** Characteristics of medical students from the University of California San Diego School of Medicine and the University of Nebraska Medical Center College of Medicine responding to a survey on self-perceived electronic health record readiness.

Characteristics		University of California San Diego (n=95), n (%)	University of Nebraska Medical Center (n=52), n (%)	Total (N=147), n (%)	*P* value^a^
**Gender**					
	Female	55 (58)	25 (48)	80 (54)	.25
**Year of training**					**<.001**
	1	26 (27)	0 (0)	47 (32)	
	2	16 (17)	30 (58)	25 (14)	
	3	29 (31)	11 (21)	40 (27)	
	4	24 (25)	11 (21)	35 (24)	
**Age (years)**					**.02**
	<25	25 (26)	27 (52)	52 (35)	
	25-27	46 (49)	15 (29)	61 (41)	
	≥28	24 (25)	10 (19)	34 (23)	
**Extracurricular EHR^b^ experience (≥1 month)**					
	Student-run free clinic	64 (67)	14 (27)	78 (53)	<.001
	Inpatient setting	45 (47)	21 (40)	66 (45)	.42
	Ambulatory clinic	62 (65)	17 (33)	79 (54)	<.001

^a^Pearson chi-square test was used to evaluate differences between the UCSD School of Medicine and the University of Nebraska Medical Center College of Medicine.

^b^EHR: electronic health record.

### Impact of Extracurricular Electronic Health Record Experience

Medical students at both UCSD and UNMC reported engagement with EHRs via extracurricular activities (Table 1). These activities were undertaken by medical students outside of their formal medical school curricula (ie, not formal clinical rotations). Examples included volunteering in student-run free clinics for underserved populations as well as volunteering in inpatient settings or ambulatory clinics. Significantly higher proportions of UCSD medical students reported having 1 month or more of EHR experience in the student-run free clinic and in ambulatory settings compared with respondents at UNMC (67% vs 53% and 65% vs 54%, respectively; *P*<.001 for both comparisons). The proportion of respondents with 1 month or more of extracurricular EHR experience in inpatient settings was similar between the 2 institutions (47% vs 40%; *P*=.42).

We specifically investigated the impact of extracurricular EHR experience on overall confidence using EHRs. Overall confidence was a single Likert score to gauge the students’ overall self-perceived confidence in using EHRs. This overall confidence score was compared between students who had less than 1 month of extracurricular EHR experience and those who had 1 month or more of extracurricular EHR experience (Table 2).

All settings (ie, free clinic, inpatient, and ambulatory) were evaluated, for the cohort overall and individually at each institution. As expected, the mean Likert scores for overall confidence were generally higher for students with longer extracurricular EHR experience at both institutions. Specifically, medical students who had longer exposures to EHR interactions in inpatient settings and ambulatory clinics reported significantly higher overall confidence in using EHRs, with average Likert scores of 3.5 or greater at both institutions. These differences did not reach statistical significance for longer exposure to the student-run free clinic. Those with less than a month of extracurricular EHR experience in any of the settings had mean Likert scores of less than 3 for overall confidence.

The effects of extracurricular activities were also evaluated in a multivariable model. The extracurricular ambulatory clinic EHR experience of 1 month or more was associated with significantly higher overall confidence using the EHR after adjusting for institution, year of training, age, and gender (average increase in the Likert score of 0.57 compared with those with <1 month of experience; *P*=.004). The effect of inpatient experience, however, was borderline significant (average increase in the Likert score of 0.38; *P*=.06). Similar to the unadjusted analysis, students with ≥1 month EHR experience in the student-run free clinic were not significantly more confident in the multivariable model (*P*=.14).

**Table 2:** Impact of extracurricular electronic health record experience on mean Likert scale score for overall confidence.

Extracurricular electronic health record experience	Overall			University of California San Diego				University of Nebraska Medical Center				
	Average rating of overall confidence^a^		*P* value	Average rating of overall confidence^a^		*P* value		Average rating of overall confidence^a^			*P* value	
	<1 month	≥1 month		<1 month	≥1 month				<1 month	≥1 month		
Student-run free clinic	2.7	3.2	.06	3.1	3.3		.55		2.9	3.2		.10
Inpatient setting	2.4	3.8	<.001	2.8	3.6		.003		2.6	3.7		<.001
Ambulatory clinic	2.3	3.5	<.001	2.9	3.6		.008		2.6	3.5		<.001

^a^Average rating of overall confidence in using electronic health record among students with <1 month versus ≥1 months of experience.

### Perceptions of Electronic Health Record Readiness Across Various Domains

The survey included 15 items asking medical students to rate their comfort level with various tasks in the EHR as well as an item to rate their overall confidence in working with EHRs. We grouped task-related items into 3 domains: (1) looking up information, (2) entering new information, and (3) medication management. We compared the mean Likert scores for self-perceived comfort or readiness for each item between the 2 institutions (Table 3).

In the domain of *looking up information*, there were no significant differences between UCSD and UNMC. Medical students at both institutions reported high levels of comfort (Likert scores >4) for looking up laboratory values and finding progress notes. Students at both institutions felt less comfortable with identifying clinical documentation errors in the EHR (mean score of 2.3 at UCSD and 2.4 at UNMC).

Compared with looking up information, medical students at both institutions felt less confident while entering new information, as no mean Likert scores exceeded 4 in this domain at either institution. Of the 8 EHR tasks included in this domain, medical students at UNMC were significantly more comfortable than medical students at UCSD with 4 of these tasks: entering a new diagnosis, updating a patient’s problem list to include a new problem, documenting immunizations in the EHR, and documenting allergies in the EHR (Table 3). UCSD medical students were significantly more comfortable with messaging other providers within the EHR (2.7 vs 1.8 at UNMC; *P*<.001). Medical students from both institutions had similar comfort levels with documenting past medical history and past social history; documenting clinical encounters using templates within the EHR; and completing documentation of notes such as progress notes, admission notes, and discharge summaries.

Medical students at both institutions reported lower comfort levels with medication management in the EHR compared with looking up information and entering new information, as no mean scores exceeded 3.5 in this domain. Although UNMC medical students had a significantly greater comfort level with entering new medication orders (3.2 vs 2.5 at UCSD; *P*=.005), there were no statistically significant differences in the remaining items, such as verifying medication orders, reviewing history and scheduled medications, and performing medication reconciliation.

In response to the item “Overall, I feel prepared to use the EHR,” medical students from both institutions endorsed a midlevel comfort score, with UCSD students having a mean score of 3.1 and UNMC students having a mean score of 3.2 (*P*=.65).

**Table 3:** Average self-reported feeling of comfort with using various components of the electronic health record by institution.

Electronic health record task^a^, mean (SD)		University of California San Diego (n=95)	University of Nebraska Medical Center (n=52)	*P* value^b^
**Looking up information**				
	Laboratory	4.0 (1.3)	4.2 (0.8)	.19
	Progress note	4.1 (1.2)	4.2 (0.9)	.44
	Clinical documentation errors	2.3 (1.3)	2.4 (1.2)	.54
**Entering new information**				
	Diagnosis	2.7 (1.3)	3.5 (1.0)	<.001
	Problem reported by patient	3.1 (1.4)	3.7 (1.1)	.007
	Immunizations	2.5 (1.3)	2.9 (1.2)	.045
	Allergies	2.9 (1.4)	3.5 (1.1)	.008
	Past medical/social history	3.4 (1.3)	3.8 (1.0)	.06
	Clinical encounter documentation using template^c^	3.8 (1.3)	3.9 (1.1)	.47
	Notes^d^	3.7 (1.4)	3.8 (1.3)	.81
	Message other providers	2.7 (1.4)	1.8 (1.2)	<.001
**Medication management**				
	Entering new medication orders	2.5 (1.4)	3.2 (1.2)	.005
	Verifying medication orders	2.4 (1.3)	2.7 (1.2)	.12
	Reviewing history and scheduled medications	3.2 (1.4)	3.4 (1.1)	.35
	Reconciliation	2.4 (1.2)	2.5 (1.1)	.89
Overall, feeling prepared to work in EHR^e^		3.1 (1.3)	3.2 (1.0)	.65

^a^Average rating of comfort level using various EHR components on a scale from 1 (very uncomfortable) to 5 (very comfortable). The full survey instrument is available in Multimedia Appendix 1.

^b^Student *t* test was used to evaluate differences between UCSD and UNMC.

^c^Documenting the clinical encounter using prespecified note templates in the EHR.

^d^Documenting notes, including history and physical examination on admission, progress notes, and discharge summaries.

^e^EHR: electronic health record.

### Comparisons of Electronic Health Record Readiness Among Preclinical Students

The primary curricular difference between the 2 institutions was that UCSD did not offer formal preclinical EHR training, whereas UNMC did. Therefore, we aggregated data from first- and second-year students to analyze perceptions of EHR readiness among preclinical students (n=131, with 77 from UCSD and 44 from UNMC). The mean Likert scores for comfort and readiness to perform various EHR tasks were compared by institution (Multimedia Appendix 2). There were no significant differences in tasks related to looking up information. Tasks where UNMC preclinical students reported significantly higher levels of readiness included entering new information, such as diagnoses (*P*<.001), problems (*P*=.002), immunizations (*P*=.007), allergies (*P*<.001), prior medical/social history (*P*=.003), and medication orders (*P*<.001). The only task where UCSD preclinical students reported greater levels of comfort than UNMC preclinical students was messaging other providers (*P*=.002). Despite differences in comfort level with individual tasks, preclinical students from the 2 institutions did not have significantly different overall levels of comfort with the EHR (*P*=.14).

### Perceptions of Electronic Health Record Readiness Among Fourth-Year Medical Students

To measure self-perceived readiness in performing EHR-related tasks at the end of undergraduate medical training, we specifically analyzed data from fourth-year medical students. For nearly all survey items, there were no significant differences between fourth-year medical students at the 2 institutions (Multimedia Appendix 3). There were significant differences for only 2 survey items: UCSD fourth-year students were significantly more comfortable with messaging other providers within the EHR (3.0 vs 1.5 at UNMC; *P*=.001) and exhibited greater overall confidence (4.2 vs 3.5 at UNMC; *P*=.04). As there were no differences in all other items and because of the relatively small sample size, the data for fourth-year medical students were combined from the 2 institutions to examine general trends among the overall cohort.

**Figure 1:**
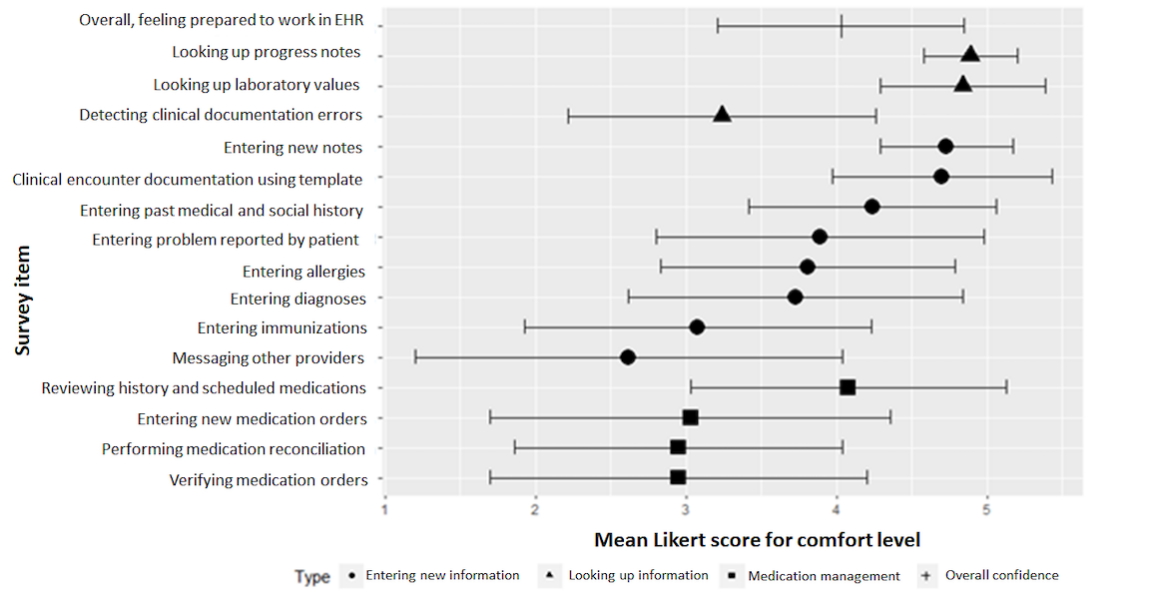
Mean Likert scores for comfort level with electronic health record–related tasks among senior fourth-year medical students from the University of California San Diego School of Medicine and the University of Nebraska Medical Center College of Medicine, 2019. EHR: electronic health record. Error bars indicate standard deviations.

Fourth-year medical students generally felt comfortable with EHR-related tasks, reporting a mean Likert score of 3 or higher for about three-fourths of the EHR-related tasks (11/15, 73%; Figure 1). Tasks for which the mean Likert scores for comfort level were less than 3 for fourth-year medical students were as follows: entering immunizations, messaging other providers, verifying medication orders, and medication reconciliation (Figure 1).

### Students’ Comments

Of the 147 respondents, 37 (25.1%) provided open-ended comments (UCSD, n=23 and UNMC, n=14). Comments from the preclinical UCSD students (15/23, 65% of all UCSD commenters) reflected the lack of formal EHR training, for example:

I have received no training.

We don’t get any training? Needs to change.

I only feel very comfortable because I have worked at Epic...Otherwise I have received very little/no training.

Overall, 2 UCSD students (9% of all UCSD commenters) mentioned exposure to EHR in the student-run free clinic, although some felt it was insufficient. For example, one third-year student wrote:

Free Clinic was good exposure but I still feel like I could have used more training on note writing before MS3.

Similarly, a first-year student wrote:

I feel very unready to work in EHR. I have to struggle through it every time I am at free clinic. Even the 4th years at free clinic struggle to help me sometimes because they are not as well-versed in EHR as they could be.

Furthermore, 5 third- and fourth-year medical students (22% of all UCSD commenters) stated that they had learned how to use the EHR through prior experience as a scribe before medical school, resident coaching while on rotations, or just “doing the work.” Several students outlined specific areas that could be addressed by training, where they felt relatively less well prepared. These areas included placing orders, more emphasis on inpatient training, and “training focused on common pitfalls or more efficient use of the EHR.”

At UNMC, 14 students provided free-text comments, evenly split between preclinical students in year 2 and clinical students in years 3 and 4. There were no comments on the lack of formal training, reflecting the structured preclinical EHR curriculum at UNMC. Moreover, 2 students (14% of all UNMC commenters) stated that this training was helpful, such as:

The allergy small group was really good.

There are so many tips and tricks that you don’t learn unless someone shows you.

However, the remaining students emphasized the importance of experiential learning over didactic training. For example, the comments included:

The EHR is a learn by doing process. I’m in the first three months of clinical rotations, and I have learned more than any of the training sessions.

I think plain old practice has made the biggest difference for me.

We can get all the “trainings” you want but if we don't actually practice what we “learn,” it’s gone by the following week.

I feel like a lot of what I know how to do is through trial and error.

Similar to UCSD medical students, several UNMC students (4/14, 29%) cited prior work experience and the critical role of residents in helping them feel comfortable with the EHR.

Furthermore, 11 students (30% of all commenters between both institutions) provided suggestions for improving training, such as the need for formal training at UCSD, interactive small group sessions, request for shorter but more frequent sessions, desire for “training focused on common pitfalls or more efficient use of the EHR,” and “training to personalize EHR.”

## Discussion

### Principal Findings

The complexity of EHRs was reflected in students’ self-perceived comfort, which varied by the specific EHR components. Across 4 years of medical school, students felt more comfortable with lower-complexity tasks such as looking up existing information and less comfortable with more complex tasks such as entering new information and medication management. Students at both institutions reported lower comfort with looking up clinical documentation errors, entering information on immunizations, reconciling medications, and messaging other providers. This discrepancy persisted even among graduating seniors, with an average comfort with looking up progress notes of 4.9 (out of 5) compared with comfort with reconciling medications of 2.8. Overall, students’ self-reported comfort with working in the EHR was 3.1 at UCSD and 3.2 at UNMC.

EHR training in medical school curricula could benefit from a combination of lower- and higher-complexity tasks. If the current training focuses primarily on navigation and data acquisition from the chart, more emphasis on information entry and medical *decision making* (ie, medication management) for medical students may improve their comfort level with these tasks. Case studies or simulated exercises, where students both look up and enter information into a training EHR environment, could be one strategy to increase comfort with higher-complexity tasks.

For medical trainees, practical experience with EHRs continues to be an important factor in becoming proficient, which has also been highlighted by national guidelines [13-16]. Mean overall confidence was generally higher among students with 1 month or more experience of extracurricular EHR compared with those with less than 1 month of experience. We asked about working or volunteering in different settings because EHR tasks and experience vary by location of usage. With the exception of a student-run free clinic, a longer experience of working in both inpatient and ambulatory settings was associated with higher average comfort of working in the EHR (3.8 vs 2.4 and 3.5 vs 2.3, respectively). The results were similar for both institutions. These effects persisted even after adjusting for other factors such as age, gender, year of training, and institution.

Although both UCSD and UNMC are similar in class size and general curriculum, there were several notable differences among students. Across all stages of training, scores of UNMC students were consistently higher than those of UCSD students on individual items, although not all items were significant. This could be because of the more structured EHR curriculum at UNMC in the preclinical years. This was reinforced by a subanalysis of preclinical students, where UNMC preclinical students expressed significantly higher levels of comfort across multiple tasks in the EHR than UCSD students. Comfort levels, however, were similar among senior fourth-year students at both UNMC and UCSD, suggesting that firsthand experience during the clinical rotation years using the EHR in the context of patient care closed this initial gap. In fact, overall, graduating UCSD students felt more prepared to work with EHR compared with UNMC students (4.2 vs 3.5; *P*=.04), despite not having any formal preclinical EHR training.

For fourth-year medical students, it is possible that tasks with mean comfort Likert scores of <3, such as for entering immunizations, messaging other providers, verifying medication orders, and medication reconciliation, were reflective of difficulty performing the task itself rather than difficulty with performing the task using the EHR. This could be because of hesitation to make permanent changes in the EHR that impact a patient’s medical record outside of the encounter under which the medical student documents.

Students’ comments emphasized the importance of practical experience, and UCSD respondents reported the lack of formal training. Despite having had a formal preclinical EHR curriculum, UNMC students still emphasized the importance of experiential training. Although the value of didactic training may not be as high as experiential training, it can still provide the foundation and basic familiarity to help students feel more comfortable and confident as they approach their clinical rotations. Students can feel anxious about transitioning to the clinical environment, as they continue to develop their medical knowledge and skills, adjust to working in a clinical environment, and learn to interact with new team members [28-30]. Thus, greater familiarity with EHR could help mitigate some anxiety inherent with this transition to clinical rotations.

As with clinical skills, mentors and, specifically, residents play a large role in students’ learning of the EHR, which was also highlighted in students’ comments. Although much of the resident-led training happens organically, there could be high variability in experience among students depending on the residents’ own familiarity with the EHR. Graduate medical education programs may consider providing formal evaluations of residents’ EHR competencies and, if needed, training.

### Limitations

Our study has several limitations, including relatively low survey response rates at both institutions and a cross-sectional design. As such, the results show associations only, and we could not evaluate causality for factors such as the institution and, thus, preclinical EHR curriculum or extracurricular experience. The survey respondents at UNMC tended to be younger than the overall student population, but the demographics of survey respondents at both institutions were consistent with the overall population. We believe that 1 month is generally sufficient to learn basic workflows in 1 setting and decided to use that time frame as a cutoff point for EHR experience. Given the importance of experience in comfort using EHRs, future studies may consider collecting detailed information on prior EHR experience. A longitudinal follow-up may help evaluate how students’ responses change as they progress through the curriculum and help determine the most impactful opportunities for EHR training. In addition, in the survey instrument, we did not collect data regarding absence from medical training, such as that for extended research projects, additional degrees, health issues, or parental leave.

### Strengths

Our study strengths included multiple institutions and participants in all 4 years of training. Both institutions had vastly different preclinical EHR training curricula. Previous studies that examined EHR use among medical students focused on the types of EHR tasks performed by medical students during their clinical clerkships [20-22,24,31]. We included medical students across the training spectrum, examining the variations in preclinical curricula. In addition, we measured medical students’ self-reported comfort rather than specific tasks they could complete in the EHR. Similar to the movement toward patient-reported outcomes rather than objective clinical outcomes in the realm of clinical research, medical education research should consider the subjective experience of medical students.

### Conclusions

Medical schools worldwide strive to continue improving the education and well-being of their students. Considering the impact of extracurricular experience on EHR readiness, we should provide more practical opportunities embedded within the preclinical curriculum rather than putting the onus on students to seek out appropriate experiences. Even student-run free clinics may be insufficient to allow all students ample clinical exposure that involves EHR practice. Some medical schools are introducing clinical exposure as formal longitudinal clerkships and introducing clinical rotations earlier in their curricula [32-36]. A combination of didactic and practical experiences combined with structured mentorship and personalization will help provide better EHR training for medical students and address this critical competency in medical education.

## Appendix

Multimedia Appendix 1 Survey instrument for assessing self-perceived readiness for using electronic health records among medical students.

Multimedia Appendix 2 Mean Likert scores for self-perceived readiness to perform various electronic health record–related tasks for preclinical (first- and second-year) medical students by institution.

Multimedia Appendix 3 Mean Likert scores for self-perceived readiness to perform various electronic health record–related tasks for senior fourth-year medical students by institution.

## Abbreviations

AAMC: Association of American Medical College
EHR: electronic health record
UCSD: University of California San Diego School of Medicine
UNMC: University of Nebraska Medical Center College of Medicine

## References

[1] Adler-Milstein J, des Roches CM, Kralovec P, Foster G, Worzala C, Charles D, Searcy T, Jha AK (2015). Electronic health record adoption in US hospitals: progress continues, but challenges persist. Health Aff (Millwood).

[2] Jha AK, Burke MF, des Roches C, Joshi MS, Kralovec PD, Campbell EG, Buntin MB (2011). Progress toward meaningful use: hospitals' adoption of electronic health records. Am J Manag Care.

[3] Jha AK, DesRoches CM, Kralovec PD, Joshi MS (2010). A progress report on electronic health records in US hospitals. Health Aff (Millwood).

[4] Henry J, Pylypchuk Y, Searcy T, Patel V (2016). Health IT Dashboard.

[5] Wachter R (2017). The Digital Doctor: Hope, Hype, and Harm at the Dawn of Medicine’s Computer Age.

[6] Tipping MD, Forth VE, O'Leary KJ, Malkenson DM, Magill DB, Englert K, Williams MV (2010). Where did the day go?-a time-motion study of hospitalists. J Hosp Med.

[7] Sinsky C, Colligan L, Li L, Prgomet M, Reynolds S, Goeders L, Westbrook J, Tutty M, Blike G (2016). Allocation of physician time in ambulatory practice: a time and motion study in 4 specialties. Ann Intern Med.

[8] Robertson SL, Robinson MD, Reid A (2017). Electronic health record effects on work-life balance and burnout within the I population collaborative. J Grad Med Educ.

[9] Poissant L, Pereira J, Tamblyn R, Kawasumi Y (2005). The impact of electronic health records on time efficiency of physicians and nurses: a systematic review. J Am Med Inform Assoc.

[10] Joukes E, Abu-Hanna A, Cornet R, de Keizer NF (2018). Time spent on dedicated patient care and documentation tasks before and after the introduction of a structured and standardized electronic health record. Appl Clin Inform.

[11] Arndt BG, Beasley JW, Watkinson MD, Temte JL, Tuan W, Sinsky CA, Gilchrist VJ (2017). Tethered to the EHR: primary care physician workload assessment using EHR event log data and time-motion observations. Ann Fam Med.

[12] Tai-Seale M, Olson CW, Li J, Chan AS, Morikawa C, Durbin M, Wang W, Luft HS (2017). Electronic health record logs indicate that physicians split time evenly between seeing patients and desktop medicine. Health Aff (Millwood).

[13] Kirch D Compliance Advisory: Electronic Health Records (EHRs) in Academic Health Centers.

[14] Hammoud MM, Dalymple JL, Christner JG, Stewart RA, Fisher J, Margo K, Ali II, Briscoe GW, Pangaro LN (2012). Medical student documentation in electronic health records: a collaborative statement from the alliance for clinical education. Teach Learn Med.

[15] (2019). The Liaison Committee on Medical Education.

[16] AAMC Store.

[17] (2015). American Medical Association.

[18] (2018). The Bulletin.

[19] American Academy of Family Physicians.

[20] Wittels K, Wallenstein J, Patwari R, Patel S (2017). Medical student documentation in the electronic medical record: patterns of use and barriers. West J Emerg Med.

[21] Virden RA, Sonnett FM, Khan AN (2019). Medical student documentation in the emergency department in the electronic health record era-a national survey. Pediatr Emerg Care.

[22] Chi J, Bentley J, Kugler J, Chen JH (2019). How are medical students using the electronic health record (EHR)?: an analysis of EHR use on an inpatient medicine rotation. PLoS One.

[23] Biagioli FE, Elliot DL, Palmer RT, Graichen CC, Rdesinski RE, Kumar KA, Galper AB, Tysinger JW (2017). The electronic health record objective structured clinical examination: assessing student competency in patient interactions while using the electronic health record. Acad Med.

[24] Foster LM, Cuddy MM, Swanson DB, Holtzman KZ, Hammoud MM, Wallach PM (2018). Medical student use of electronic and paper health records during inpatient clinical clerkships: results of a national longitudinal study. Acad Med.

[25] Rajaram A, Hickey Z, Patel N, Newbigging J, Wolfrom B (2020). Training medical students and residents in the use of electronic health records: a systematic review of the literature. J Am Med Inform Assoc.

[26] Sullivan GM, Artino AR (2013). Analyzing and interpreting data from likert-type scales. J Grad Med Educ.

[27] R-Project.

[28] Wenrich M, Jackson MB, Scherpbier AJ, Wolfhagen IH, Ramsey PG, Goldstein EA (2010). Ready or not? Expectations of faculty and medical students for clinical skills preparation for clerkships. Med Educ Online.

[29] Small RM, Soriano RP, Chietero M, Quintana J, Parkas V, Koestler J (2008). Easing the transition: medical students' perceptions of critical skills required for the clerkships. Educ Health (Abingdon).

[30] Haglund ME, aan het Rot M, Cooper NS, Nestadt PS, Muller D, Southwick SM, Charney DS (2009). Resilience in the third year of medical school: a prospective study of the associations between stressful events occurring during clinical rotations and student well-being. Acad Med.

[31] White J, Anthony D, WinklerPrins V, Roskos S (2017). Electronic medical records, medical students, and ambulatory family physicians: a multi-institution study. Acad Med.

[32] Gheihman G, Jun T, Young GJ, Liebman D, Sharma K, Brandes E, Ogur B, Hirsh DA (2018). A review of longitudinal clinical programs in US medical schools. Med Educ Online.

[33] Latessa R, Beaty N, Royal K, Colvin G, Pathman DE, Heck J (2015). Academic outcomes of a community-based longitudinal integrated clerkships program. Med Teach.

[34] Royan R, Wu C, Theyyunni N, Montas S, Cranford J, House J, Lukela M, Santen S (2018). Anything but shadowing! Early clinical reasoning in emergency department improves clinical skills. West J Emerg Med.

[35] Whipple ME, Barlow CB, Smith S, Goldstein EA (2006). Early introduction of clinical skills improves medical student comfort at the start of third-year clerkships. Acad Med.

[36] Kossoff EH, Hubbard TW, Gowen CW (1999). Early clinical experience enhances third-year pediatrics clerkship performance. Acad Med.

