# Supplementary material for: Self-Perceptions of Readiness to Use Electronic Health Records Among Medical Students: Survey Study
Source: JMIR Med Educ. 2020 Jun 12;6(1):e17585. doi: 10.2196/17585 (PMC7320310; doi:10.2196/17585)
Supplement: Multimedia Appendix 1 [file mededu_v6i1e17585_app1.pdf]

Default Question Block

Which program are you enrolled in?

- Pharmacy
- Medicine

Which program year are you currently enrolled in? (as of January 2019)

- 1st year
- 2nd year
- 3rd year
- 4th year

Gender

- Male
- Female

Age

- <25
- 25-27
- 28-30
- >30

How much experience do you have with the following electronic health records (outside of the school's curriculum)?

	No experience	<1 month	1-3 months	4-6 months	>6 months
Community Pharmacy	○	○	○	○	○
Ambulatory Clinic	○	○	○	○	○
UCSD Free Clinic	○	○	○	○	○
Inpatient Setting	○	○	○	○	○

Rate how comfortable you feel doing the following on a scale from 1 to 5, with 1 being very uncomfortable and 5 being very comfortable.

	1 (Very Uncomfortable)	2	3	4	5 (Very Comfortable)
Finding laboratory results	○	○	○	○	○
Finding progress notes	○	○	○	○	○
Identifying clinical documentation errors in the electronic health record	○	○	○	○	○
Entering a new diagnosis	○	○	○	○	○
Reviewing medication administration history and future scheduled medications	○	○	○	○	○
Performing medication reconciliation in the electronic health record	○	○	○	○	○
	1 (Very Uncomfortable)	2	3	4	5 (Very Comfortable)
Updating a patient's problem list to include a new problem	○	○	○	○	○
Documenting [illegible]					

	1 (Very Uncomfortable)	2	3	4	5 (Very Comfortable)
Documenting immunizations in the electronic health record	○	○	○	○	○
Documenting allergies in the electronic health record	○	○	○	○	○
Documenting a past medical history and a social history (smoking, dietary, illicit drugs, etc.) in the electronic health record	○	○	○	○	○
Documenting a clinical encounter using templates or SOAP notes in the electronic health record	○	○	○	○	○
Documenting notes, including: history and physical on admission, progress notes, discharge summaries	○	○	○	○	○
	1 (Very Uncomfortable)	2	3	4	5 (Very Comfortable)
Entering medication orders in the electronic health record	○	○	○	○	○
Verifying medication orders in the electronic health record	○	○	○	○	○
Messaging other providers within the electronic health record	○	○	○	○	○
Overall, I feel prepared to use the electronic health record	○	○	○	○	○

Comment on your EHR training/preparedness to work in EHR:

Powered by Qualtrics
