# Supplementary material for: Self-Perceptions of Readiness to Use Electronic Health Records Among Medical Students: Survey Study
Source: JMIR Med Educ. 2020 Jun 12;6(1):e17585. doi: 10.2196/17585 (PMC7320310; doi:10.2196/17585)
Supplement: Multimedia Appendix 2 [file mededu_v6i1e17585_app2.docx]

**Multimedia Appendix 2. Mean Likert scores for self-perceived readiness to perform various EHR-related tasks for pre-clinical (first and second year) medical students by institution.**

| **EHR Task^a^** | **UCSD** (n=77) | **UNMC** (n=44) | ***P*-value^b^** |
| --- | --- | --- | --- |
| **Looking up information** |  |  |  |
| Lab | 3.8 | 4.1 | .15 |
| Progress Note | 3.8 | 4.1 | .30 |
| Clinical documentation errors | 2.0 | 2.3 | .16 |
| **Entering new information** |  |  |  |
| Diagnosis | 2.5 | 3.4 | <.001 |
| Problem reported by patient | 2.9 | 3.6 | .002 |
| Immunizations | 2.3 | 3.0 | .007 |
| Allergies | 2.6 | 3.5 | <.001 |
| Past medical/ social history | 3.1 | 3.8 | .003 |
| Clinical encounter documentation using template^c^ | 3.5 | 3.8 | .36 |
| Notes^d^ | 3.4 | 3.6 | .49 |
| Message other providers | 2.7 | 1.9 | .002 |
| **Medications** |  |  |  |
| Entering new medication orders | 2.3 | 3.3 | <.001 |
| Verifying medication orders | 2.3 | 2.7 | .07 |
| Reviewing history and scheduled medications | 2.9 | 3.3 | .08 |
| Reconciliation | 2.3 | 2.5 | .34 |
| **Overall, feeling prepared to work in EHR** | 2.8 | 3.1 | .14 |

Abbreviations: EHR= electronic health record, UCSD = University of California San Diego, UNMC = University of Nebraska Medical Center.

^a^Average rating of comfort level using various EHR components on a scale from 1 (very uncomfortable) to 5 (very comfortable). Full survey instrument available in Appendix 1.

^b^Student’s t-tests were used to evaluate differences between UCSD and UNMC.

^c^Documenting the clinical encounter using pre-specified note templates in the EHR.

^d^Documenting notes, including: history and physical on admission, progress notes, and discharge summaries.
